# Primitive erythrocytes are generated from hemogenic endothelial cells

**DOI:** 10.1038/s41598-017-06627-9

**Published:** 2017-07-25

**Authors:** Monika Stefanska, Kiran Batta, Rahima Patel, Magdalena Florkowska, Valerie Kouskoff, Georges Lacaud

**Affiliations:** 1 Cancer Research UK Stem Cell Biology Group, Cancer Research UK Manchester Institute, The University of Manchester, Wilmslow road, Manchester, M20 4BX UK; 2 Division of Developmental Biology & Medicine, The University of Manchester, Michael Smith Building, Oxford Road, Manchester, M13 9PT UK

## Abstract

Primitive erythroblasts are the first blood cells generated during embryonic hematopoiesis. Tracking their emergence both *in vivo* and *in vitro* has remained challenging due to the lack of specific cell surface markers. To selectively investigate primitive erythropoiesis, we have engineered a new transgenic embryonic stem (ES) cell line, where eGFP expression is driven by the regulatory sequences of the embryonic *βH1* hemoglobin gene expressed specifically in primitive erythroid cells. Using this ES cell line, we observed that the first primitive erythroblasts are detected *in vitro* around day 1.5 of blast colony differentiation, within the cell population positive for the early hematopoietic progenitor marker CD41. Moreover, we establish that these eGFP^+^ cells emerge from a hemogenic endothelial cell population similarly to their definitive hematopoietic counterparts. We further generated a corresponding βH1-eGFP transgenic mouse model and demonstrated the presence of a primitive erythroid primed hemogenic endothelial cell population in the developing embryo. Taken together, our findings demonstrate that both *in vivo* and *in vitro* primitive erythrocytes are generated from hemogenic endothelial cells.

## Introduction

Primitive erythroblasts are the first blood cells that are formed during embryogenesis^1^. These cells differ from definitive erythrocytes, generated later, by several features such as their size, presence of nuclei, oxygen carrying potential and gene expression pattern^2^. During murine embryogenesis, primitive erythroid (Ery/P) progenitors appear in the yolk sac blood islands around E7.25^3^ within a first wave of hematopoiesis that also generates macrophages and megakaryocytes^4, 5^. All subsequent waves of blood emergence in the embryo, from E8.25 onward, are defined as definitive hematopoiesis. This includes erythro-myeloid progenitors (EMPs) produced in the yolk sac which give rise to definitive erythrocytes, macrophages, megakaryocytes and other myeloid lineages as well as T and B progenitors produced in the yolk sac and para-aortic splanchnopleura and HSCs produced in the dorsal aorta, vitelline and umbilical arteries. Ery/P progenitor cells are produced for only 2 days during ontogeny^6^ as a wave of maturing erythroblasts that provide the rapidly growing embryo with a sufficient amount of oxygen to support growth and survival until the production of definitive erythrocytes. Nevertheless, it has been demonstrated that even after birth, low frequencies of mature primitive erythrocytes are still present^7^. Although it was initially thought that primitive erythrocytes remain nucleated, it has been more recently established that they enucleate between day 12.5 and 16.5 of gestation^7^.

A mesodermal progenitor – the hemangioblast, has been demonstrated to give rise *ex vivo* to both primitive and definitive hematopoietic, endothelial and vascular smooth muscle lineages^8, 9^. During embryonic stem cells (ESCs) differentiation, the equivalent of this precursor, the blast colony forming cell (BL-CFC), generates colonies with precursors for both primitive and definitive hematopoietic cells^10^. In this context, definitive blood cells were defined as cells of all blood lineages, including definitive erythroid, myeloid and lymphoid cells, with the exception of primitive erythroid cells. Indeed if defining primitive hematopoiesis in the embryo is straightforward since this wave is restricted in time and space, identifying a primitive wave during the *in vitro* differentiation of ESCs is more challenging. Only primitive erythroid precursors can be identified with certainty as being part of this primitive wave, while it is difficult to distinguish macrophages and megakaryocytes that can have been either generated from primitive or definitive hematopoiesis^4, 11, 12^.

Definitive hematopoietic cells were shown to be generated from BL-CFC through an intermediate cell population of specialised endothelium, i.e. from an hemogenic endothelium^13–15^. Accordingly, definitive TER119^+^ erythrocytes were shown to emerge *in vivo* from endothelial cells^16^. The cellular precursor for primitive erythroid cells remains much less characterised. It is still not yet established if primitive erythroid cells directly emerge from hemangioblast (BL-CFCs) or if they are generated through a hemogenic endothelium intermediate. Supporting, the hypothesis that these cells are generated from a hemogenic endothelium, primitive erythroid progenitors were shown to be enriched in mesodermal cell populations positive for TIE2 and PECAM-1 endothelial markers^17^. Furthermore, ε-globin H2B-EGFP positive cells were found within FLK1 and VE-CADHERIN double positive cell population^18^.

Studies on primitive erythropoiesis have been hampered by the difficulty in accessing the yolk sac blood islands and by the absence of specific cell surface markers for this lineage. Furthermore, primitive erythropoiesis is an extremely rapid developmental process and the number of cells per embryo is limited. Transgenic ES lines and mouse reporter models have been instrumental to study primitive erythropoiesis at both the molecular and cellular levels. Indeed, two transgenic mouse models have been previously generated to investigate primitive erythropoiesis. In the first one, human embryonic *ε*-globin gene promoter was coupled with jellyfish KGFP fluorescent protein^19^. This mouse model was used to characterize the expression of cell surface markers by primitive erythrocytes between day 9.5 and 14.5 of embryogenesis as well as their enucleation. The same research group generated a second mouse model in which the fluorescent protein was replaced by H2B-EGFP fusion^20^. This animal model was used to study primitive erythrocyte maturation within the fetal liver^20^. In the present study, we hypothesized that βH1-globin, whose expression precedes that of the *εy-globin* gene^21^ might represent a more suitable marker to track the onset of primitive erythropoiesis and to conclusively establish the cellular origin of primitive erythrocytes.

Here, we report the generation and validation of a transgenic ES cell line where eGFP is driven by the embryonic hemoglobin βH1 regulatory sequences, to track the emergence of primitive erythrocytes *in vitro*. With this ES cell line we demonstrate that primitive erythroid progenitors appear around day 1.5 of BL-CFC development *in vitro* within the CD41 positive cell population. The culture of isolated hemogenic endothelial cells demonstrates their primitive erythroid potential. We further demonstrate the presence of a similar hemogenic endothelial cell population committed to primitive erythropoiesis in developing embryos. Altogether, our study establishes that similar to other blood lineages, the generation of primitive erythrocytes also goes through a hemogenic endothelium intermediate stage.

## Results

### Expression of *βH1-eGFP* marks the first committed primitive precursors

**Figure 1:**
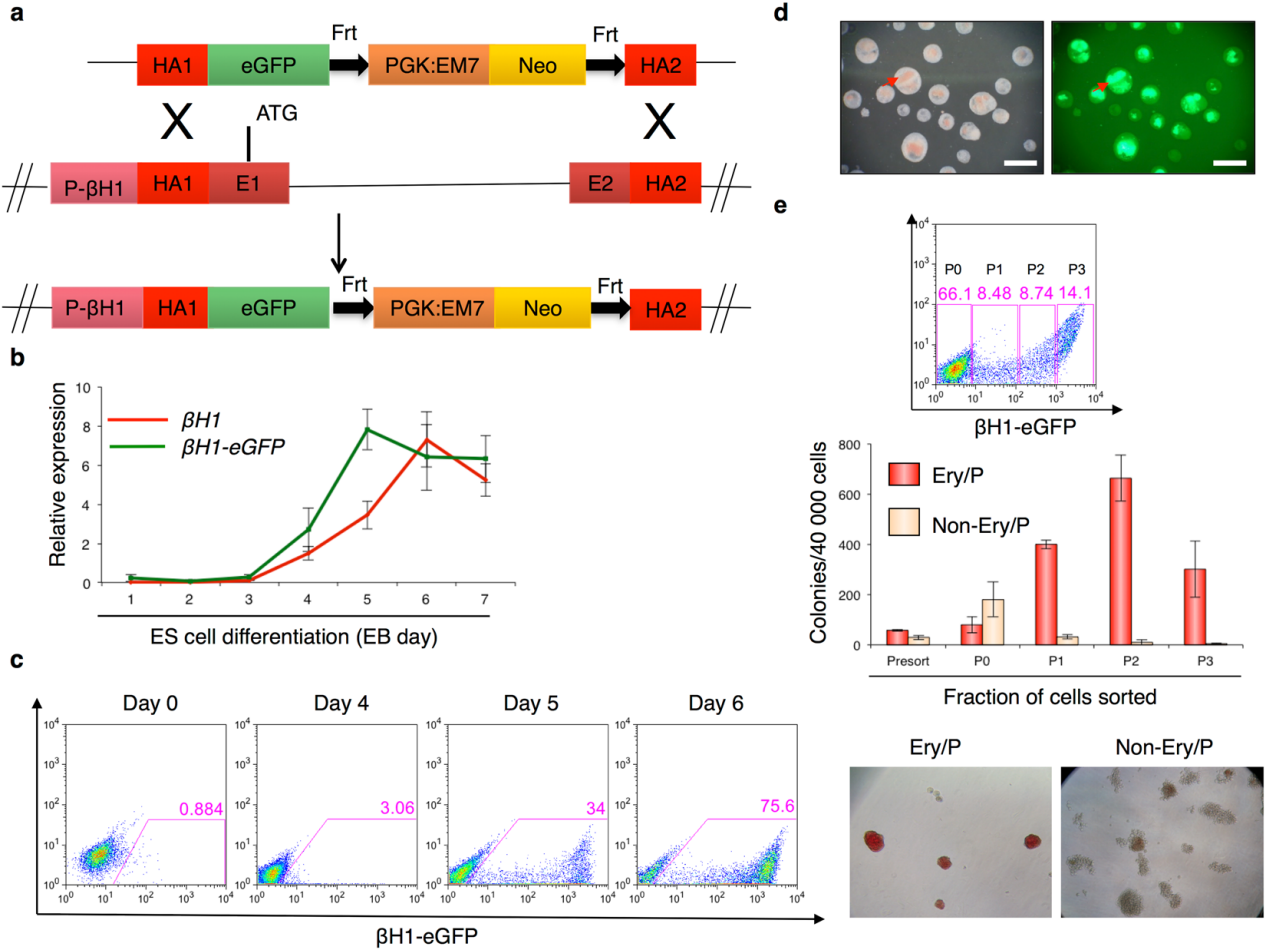
Expression of *βH1-eGFP* marks the first committed primitive precursors: (**a**) Schematic representation of the strategy used to generate βH1-eGFP BAC. A targeting vector containing two homology arms amplified from the βH1 BAC (bMQ433i10), eGFP cDNA, PGK promoter, EM-7 promoter, and Neomycin resistance gene flanked by Frt sites was generated and linearized. The initiation codon (ATG) is localized within exon 1. Two homology arms were designed on the BAC and amplified. HA - homology arm; E- exon; Neo - Neomycin, P-βH1 - promoter βH1 (**b**) Gene expression analyses of endogenous *βH1* and transgenic *βH1-eGFP* with respect to *β-actin* during EB differentiation from day 1 until day 7. Data represented are mean ± SEM (n = 3). (**c**) FACS analysis showing the expression of βH1-eGFP at indicated days during EB differentiation. (**d**) Bright field and fluorescent images of day 7 EBs. Arrows mark the hemoglobinised EBs and high expression levels of βH1-eGFP. Scale bar 300 μM. **(e)** FACS analysis of βH1-eGFP expression levels in EBs at day 5 (top panel). Populations expressing different levels of βH1-eGFP were sorted (P0-P3). Day 5 EBs prior to sorting was used as control. Number of Ery/P or non-Ery/P CFUs observed following replating of 40,000 sorted cell population (Middle panel). Data represented are mean ± SEM (n = 3). Bright field images of representative Ery/P and non-Ery/P hematopoietic colonies (Bottom panel).

To track the development of primitive erythrocytes, we engineered a *βH1*- transgenic reporter ES cell line. A BAC (bMQ433i10) containing the full *β globin* locus was modified by recombineering to insert an eGFP construct followed by a neomycin resistance gene expression cassette in the *Hbb-βH1* gene. As a result of recombineering, the fluorescent eGFP expression is driven by the regulatory sequences of the *βH1* gene (Fig. 1a and Supplementary Fig. S1a). The modified BAC was then electroporated into ES cells and several clones were selected with geneticin (G418). In order to determine whether the expression of the *βH1-eGFP* reporter gene correlates with endogenous *βH1* gene, transgenic ES clones were differentiated as embryoid bodies (EBs) for 7 days. We monitored the expression of the transgenic *βH1-eGFP* and endogenous *βH1* at different days of differentiation process. As shown in Fig. 1b for one representative ES cell clone, the expression of transgene *βH1-eGFP*, detected by real time PCR, was strongly correlated with the endogenous *βH1* gene expression. In both cases, expression was detected from day 4 of differentiation and levels increased over the course of differentiation. These results indicate that the transgenic *βH1-eGFP* reporter ES cell line recapitulates the expression of endogenous *βH1* and therefore *eGFP* expression may be used to qualitatively track primitive erythropoiesis. The presence of the eGFP fluorescent protein was also evaluated by flow cytometry during the time course of ES cell differentiation (Fig. 1c and Supplementary Fig. S1b). At day 4 of EB differentiation, around 3% of cells expressed low levels of eGFP and the percentage of positive cells reached 75% by day 6 (Fig. 1c). By day 7 of differentiation, the EBs were highly hemoglobinised and these red regions displayed high levels of eGFP (Fig. 1d), further indicating a correlation between eGFP expression and primitive erythropoiesis.

To confirm that eGFP expression reflects the emergence of primitive erythroid precursors, cells from day 5 EBs were sorted based on their level of eGFP expression: negative (P0, geometric MFI: 3.03), low (P1, geometric MFI: 26.8), intermediate (P2, geometric MFI: 390) and high (P3, geometric MFI: 2113) (Fig. 1e top panel) and replated in semi-solid hematopoietic medium (Supplementary Fig. S1c). As shown in Fig. 1e (middle panel), βH1-eGFP positive cell populations (P1–P3) mostly gave rise to typical bright red circular primitive erythroid (Ery/P) colonies. In contrast, the βH1-eGFP negative (P0) cell population mainly generated other types of hematopoietic colonies. Given the absence of absolute markers to conclusively distinguish between primitive or definitive macrophages or megakaryocytes that could both be present at this early stage, we will refer to these colonies as non-Ery/P instead of primitive or definitive hematopoietic. The generation of few non-Ery/P hematopoietic colonies from the P1 (βH1-eGFP low) cell population might reflect a potential contamination by the P0 cell population (Supplementary Fig. S1d). Representative images of primitive erythroid and non-Ery/P colonies are shown in Fig. 1e (bottom panel). To confirm the primitive nature of the erythroid colonies generated by βH1-eGFP positive cells, we cultured day 5 EB sorted βH1-eGFP positive cells in primitive (Erythropoietin only) or definitive (Erythropoietin + Kit-Ligand) erythroid promoting culture conditions. We did not observe any striking increase in the numbers of non-Ery/P colonies in definitive culture conditions indicating that this cell population does not contain a large contamination of definitive erythroid progenitors (Supplementary Fig. S2a and b). Furthermore, O-dianisidine and May-Grunwald Giemsa staining of cytospinned cells grown in both culture conditions confirmed the presence of large nucleated hemoglobinised cells (Supplementary Fig. S2c) consistent with primitive erythrocytes, whereas small enucleated hemoglobinised cells were observed upon staining of isolated non-Ery/P colonies (Supplementary Fig. S2d). In addition, PCR analyses confirmed the expression of embryonic globins by the cells generated from day 5 EB sorted βH1-eGFP positive cells (Supplementary Fig. 2e). The expression of adult globins was not significantly increased in definitive erythroid promoting culture conditions eliminating again the possibility of the large presence of definitive erythroid progenitors in this fraction. Altogether these data confirm the primitive erythroid nature of the bright red circular colonies generated by βH1-eGFP^+^ cells. This indicates that the eGFP positive cell subpopulations are mainly devoid of non-Ery/P hematopoietic progenitors and contain essentially primitive erythroid (Ery/P) progenitors.

### Tracking the emergence of primitive erythrocytes during hemangioblast development

**Figure 2:**
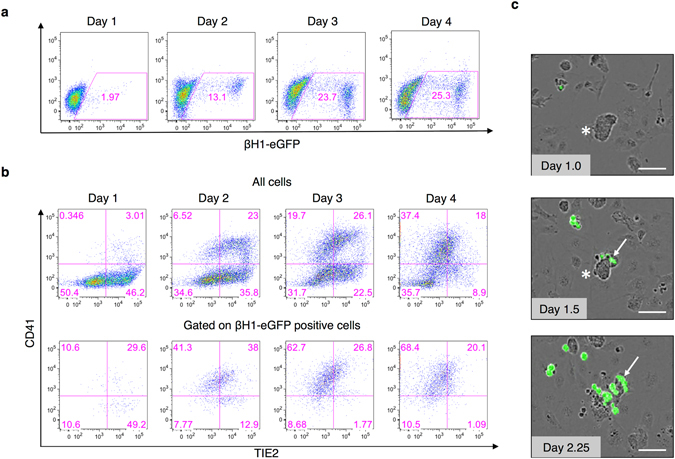
βH1-eGFP expression in the context of hemangioblast development *in vitro*. BL-CFCs were sorted from day 3.25 EBs and were further cultured in blast differentiation medium. FACS analyses showing the expression of βH1-eGFP (**a**), TIE2 and CD41 ((**b**), top panel) during blast colony differentiation at indicated days. Cells expressing βH1-eGFP were analysed for expression of TIE2 and CD41 ((**b**), bottom panel). (**c**) Phase contrast images of BL-CFC differentiation at indicated days. Cells positive for βH1-eGFP (marked with an arrow) appears within the core (marked with an asterisk). Scale bar 100 μm.

To define the pattern of emergence of primitive erythroid committed precursors during blast colony development *in vitro*, ES cells were first differentiated as EBs and then cells from day 3.25 EBs were dissociated and sorted based on the expression of the mesodermal marker FLK1 to enrich for BL-CFCs. FLK1 cells were then cultured for 4 days and analysed by FACS for the expression of cell surface markers. As depicted in Fig. 2a, the first cells expressing βH1-eGFP (1.97%) appeared already at day 1 of the FLK1 culture. Their numbers increased rapidly from day 2 onwards and reached over 25% by day 4 of blast colony differentiation *in vitro* (Supplementary Fig. S3a). Previous reports have established that the generation of blood cells during blast cultures proceed through the upregulation of TIE2 followed by the acquisition of a hematopoietic immuno-phenotype manifested by CD41 expression and down-regulate TIE2^13^. We therefore analysed the expression of both endothelial (TIE2) and hematopoietic (CD41) markers either in all the cells present or specifically in the βH1-eGFP expressing population (Fig. 2b). Most βH1-eGFP positive cells expressed either TIE2 and/or CD41. Later on, when TIE2 was down-regulated, βH1-eGFP positive cells were mostly found within the cell population marked by the expression of CD41 (Fig. 2b and Supplementary Fig. S3b). To further visualize and investigate the emergence of βH1-eGFP positive cells during *in vitro* blast colony differentiation, time-lapse imaging was performed every 30 minutes, acquiring both bright field and fluorescence. At day 1.0 of blast colony differentiation clusters of tight adherent cells were observed (Fig. 2c and Supplementary Video 1). Few hours later, at day 1.5 of BL-CFC differentiation the first cells expressing βH1-eGFP were seen to emerge from these tight clusters. These βH1-eGFP positive cells then proliferated during the course of the culture (Fig. 2c and Supplementary Video 1). Altogether, these results indicate that the first primitive erythrocytes tracked by βH1-eGFP expression emerge early during blast colony differentiation *in vitro* and are mostly positive for TIE2 and CD41 expression.

### Primitive erythrocytes emerge from hemogenic endothelial populations *in vitro*

**Figure 3:**
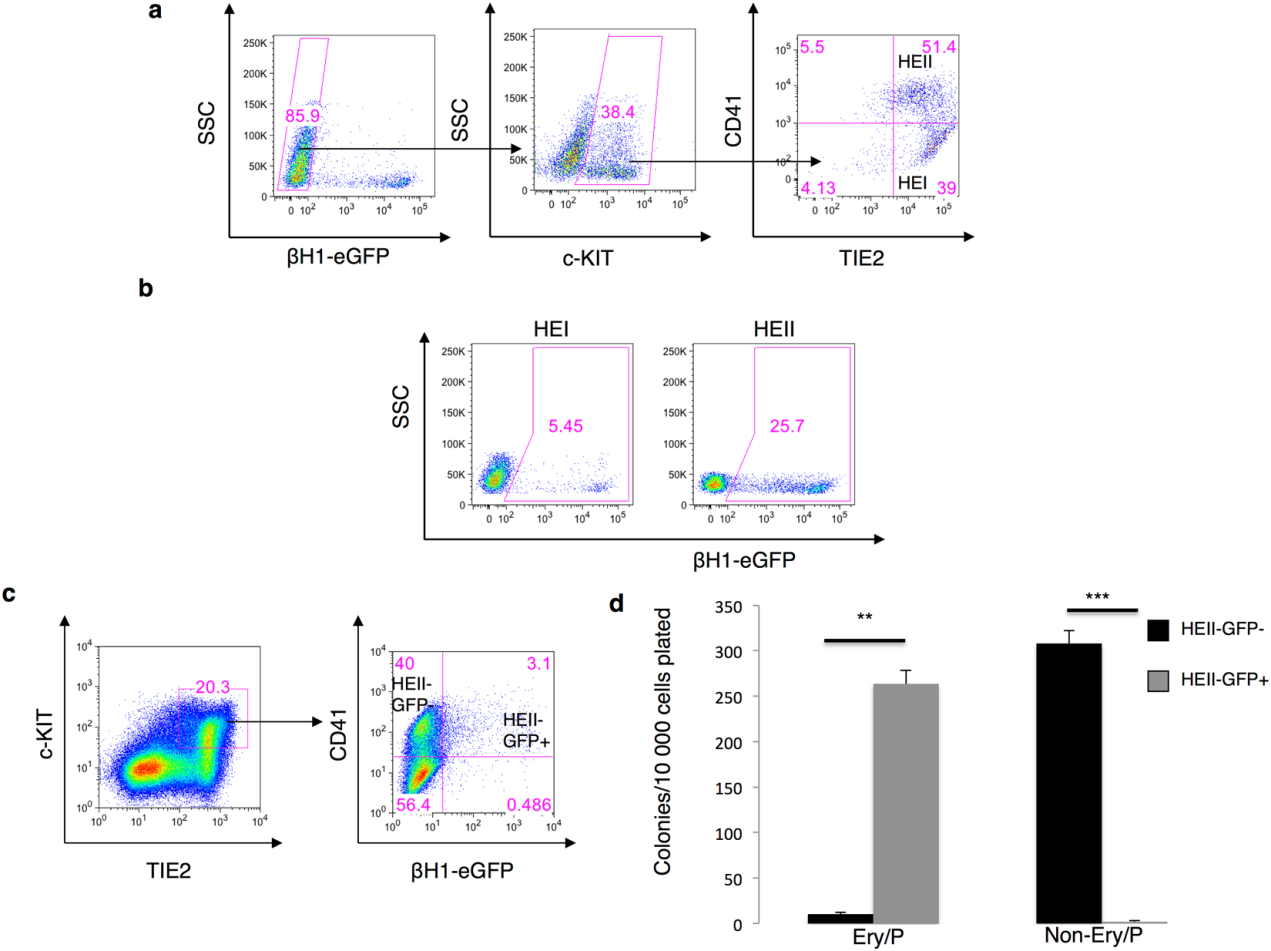
Blast colony forming cells expressing TIE2, c-KIT, CD41 and βH1-eGFP have primitive hematopoietic potential. (**a**–**c**) BL-CFCs were sorted from day 3.25 EBs and further cultured in liquid blast media for 42 hours. (**a**) Gating strategy used to isolate different cell populations of hemogenic endothelial (HE) I and HEII negative for βH1-eGFP and based on TIE2, c-KIT and CD41 cell surface markers from liquid blast cultures. (**b**) FACS analysis of the expression of βH1-eGFP in HEI and HEII populations in liquid blast cultures. **(c)** Gating strategy used to isolate different cell populations based on TIE2, c-KIT, CD41 and βH1-eGFP markers from liquid blast cultures (**d**) Number of Ery/P and non-Ery/P observed per following replating of 10,000 HEII GFP^+^ and HEII GFP^−^ sorted cell populations (From Fig. 3c). Data represented are mean ± SEM (n = 3). **p < 0.01 ***p < 0.001.

Definitive hematopoietic cells are generated *in vivo* from a unique endothelial population called hemogenic endothelium^22–24^. Similarly hemangioblasts give rise to definitive hematopoietic precursors *via* a common hemogenic endothelial cell stage^13^. However, whether primitive erythropoiesis occurs *via a* hemogenic endothelial intermediate has never been directly established. In order to investigate this, hemogenic endothelial cells were generated from βH1-eGFP ES cells. For this, ES cells were first differentiated as EBs and FLK1 positive cells were then sorted by day 3 and cultured in liquid blast media for two additional days. The cells were then harvested by trypsinization and cell populations representing different successive stages of the transition between hemogenic endothelium and hematopoietic cells isolated based the expression of the endothelial marker TIE2, the hematopoietic and endothelial progenitor marker c-KIT and the hematopoietic progenitor marker CD41 (Fig. 3a and Supplementary Fig. S4a). The two different cell populations isolated were hemogenic endothelium type I fraction (HEI defined as TIE2^+^, c-KIT^+^ and CD41^−^) and hemogenic endothelium type II subpopulation (HEII defined as TIE2^+^, c-KIT^+^ and CD41^+^). The sorted cells were then cultured in hemogenic endothelium culture conditions for 48 hours. As shown in Fig. 3b, both the HEI and HEII hemogenic endothelial cell populations were able to generate *de novo* βH1-eGFP positive cells. The frequency of primitive erythrocytes generated by the HEII cell population was higher than that generated by HEI cells (Fig. 3b and Supplementary Fig. S4b). In order visualize the emergence of βH1-eGFP positive cells from hemogenic endothelial cells, βH1-eGFP negative HE cells were sorted from day 5 EBs and further cultured on gelatin-coated plates. As shown in Supplementary Fig. S4c, the emergence of βH1-GFP positive cells was readily observed within two days of culture. These results demonstrate that primitive erythroid cells, marked by the expression of βH1-eGFP are readily generated from hemogenic endothelial cell populations.

**Figure 4:**
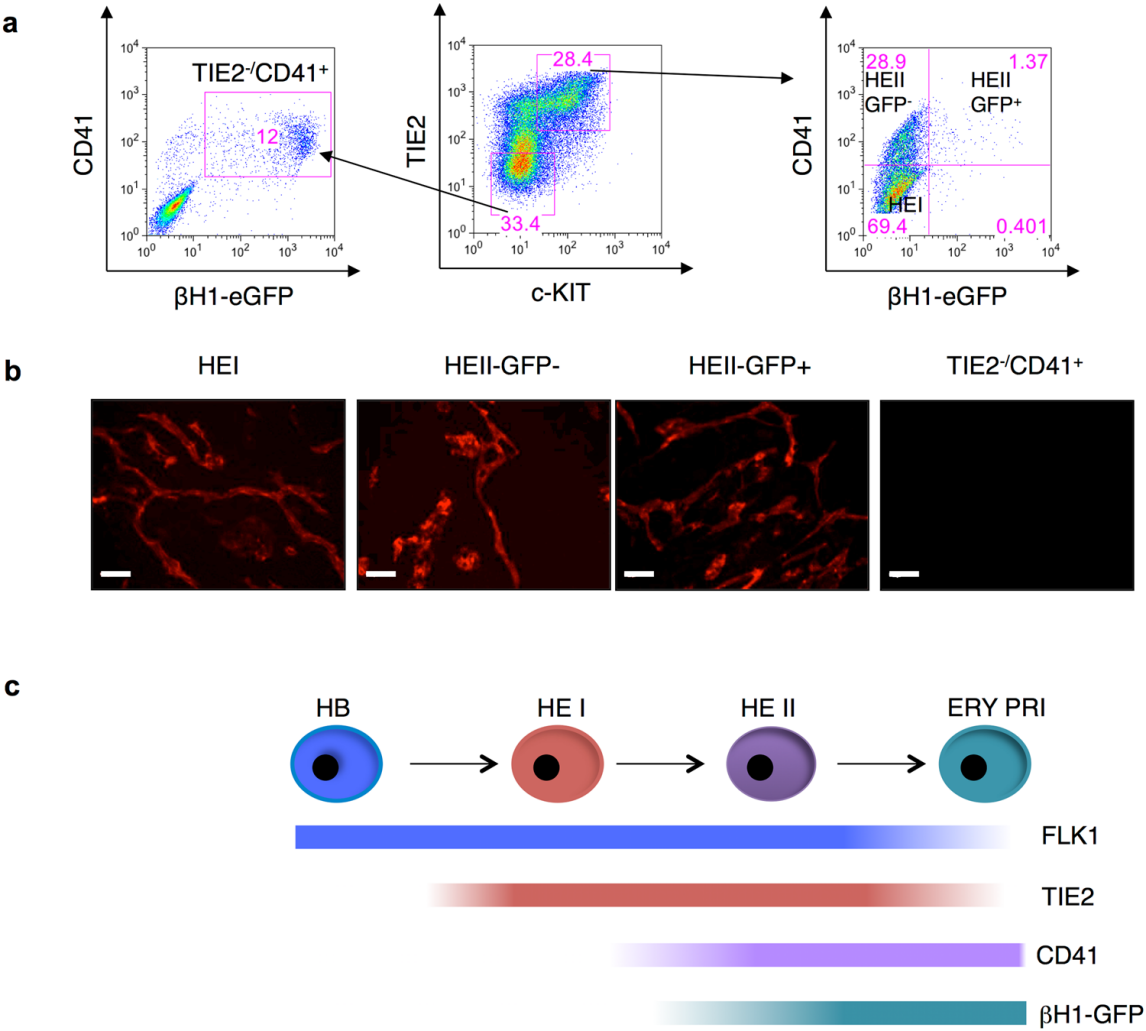
Endothelial potential of cells expressing TIE2, c-KIT, CD41 and βH1-eGFP. (**a**,**b**) BL-CFCs were sorted from day 3.25 EBs and further cultured in liquid blast media for 42 hours. (**a**) Gating strategy used to isolate different cell populations erythroid primitive (TIE2^−/^CD41^+^) HEI, HEII GFP^−^ and HEII GFP^+^ based on TIE2, c-KIT, CD41 and βH1-eGFP markers. (**b**) Fluorescent microscopy images of endothelial networks stained with CD31-PE antibody generated from indicated populations cultured on OP9 stromal cells for 14 days. TIE2^−^CD41^+^ population did not form the endothelial structures. Scale bar 500 μm. (**c**) Schematic diagram representing the basic developmental stages of BL-CFC differentiation. The expression of βH1-eGFP is shown in the context of other markers.

Our FACS analyses during blast colony development indicated that the first emerging βH1-eGFP^+^ cells expressed TIE2 and could therefore still be endothelial cells (Fig. 2b). Indeed FACS analyses indicated that a fraction of the HEII population express βH1-eGFP (Fig. 3c and Supplementary Fig. S4d). To determine if these cells have already acquired the potential to generate primitive erythroid colonies, prior to losing their positivity for TIE2, the two fractions of HEII expressing or not βH1-eGFP (TIE2^+^ c-KIT^+^ CD41^+^ βH1-eGFP^−^ and TIE2^+^ c-KIT^+^ CD41^+^ βH1-eGFP^+^) were sorted and directly replated into semi-solid clonogenic assay. As shown in Fig. 3d, the HEII GFP^+^ population almost exclusively gave rise to primitive erythroid colonies. In contrast the HEII GFP^−^ cell population generated mostly non-Ery/P and only a small number of Ery/P colonies. Altogether this data suggest that the fraction of this cell population expressing βH1-eGFP represents the first committed primitive erythroid cells generated *in vitro* during ES cell differentiation. To confirm that these two HEII cell populations still had an endothelium potential, they were replated onto OP9 stroma in MEM-α medium for 12 days (Fig. 4a) and then stained with the endothelial marker CD31 (PECAM-1) antibody (Fig. 4b). Both fractions of HEII cell populations (HEII GFP^−^ and HEII GFP^+^) generated endothelial structures. These structures were also generated by cells from the HEI population, but were absent from the cultures initiated with CD41^+^ hematopoietic cells. These findings strongly support the hypothesis that these TIE2^+^ c-KIT^+^ CD41^+^ βH1-eGFP^+^ cells represent a hemogenic endothelial cell population primed for primitive erythroid potential. Altogether these results establish that βH1-eGFP^+^ cells emerge within the HEII population generated upon the upregulation of CD41 from the HEI population (Fig. 4c).

### Primitive erythrocytes emerge from hemogenic endothelial populations *in vivo*

**Figure 5:**
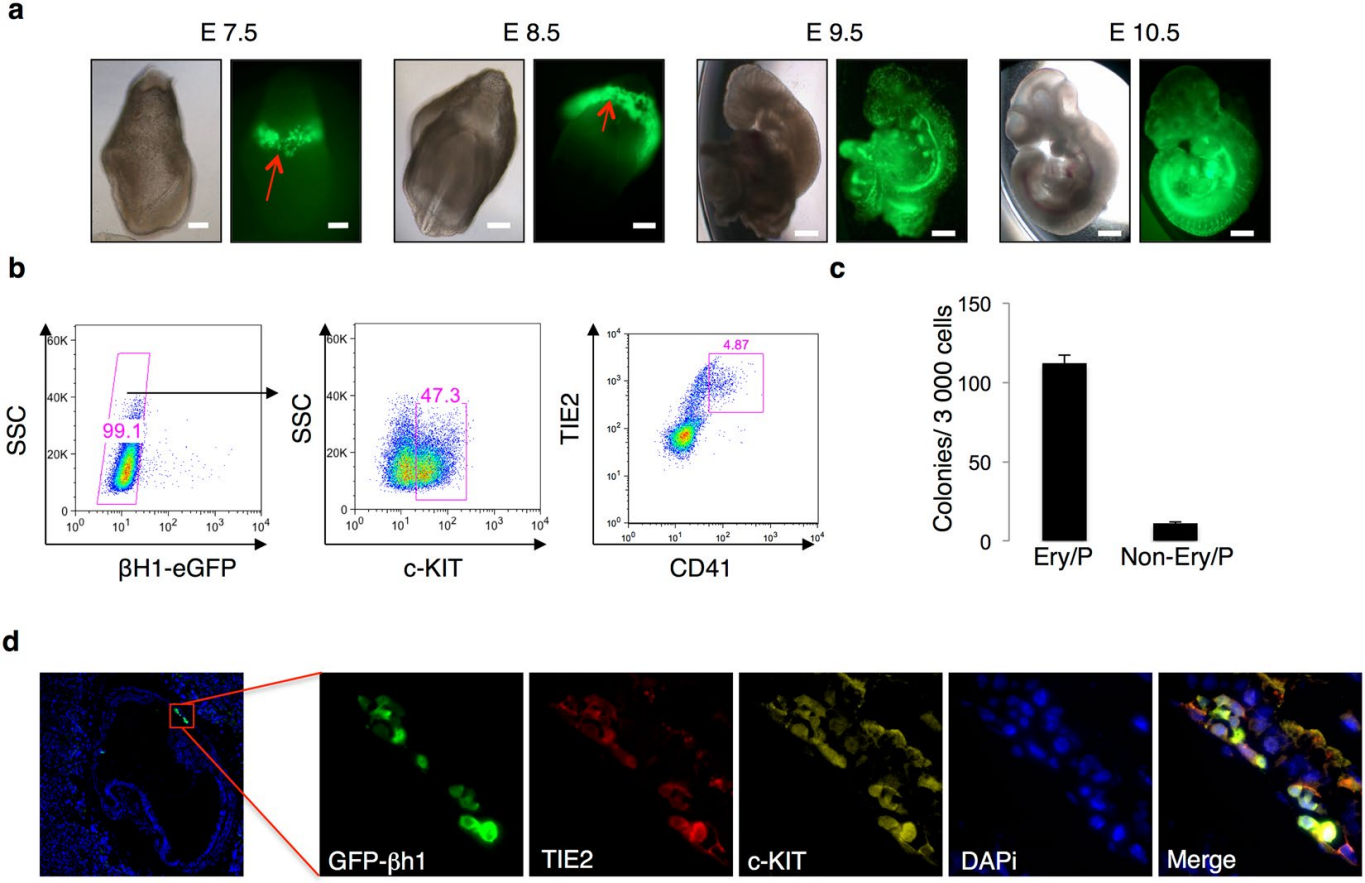
Primitive erythrocytes are generated from hemogenic endothelial cells *in vivo*. (**a**) Phase contrast and fluorescent images showing representative βH1-eGFP transgenic embryos at days E7.5, E8.5, E9.5 and E10.5. The blood island region in yolk sac is marked with an arrow in E 7.5 and 8.5 images. Scale bar 100 μm. (**b**) Gating strategy used to isolate hemogenic endothelial cell population, βH1-eGFP^−^, c-KIT^+^, TIE2^+^ and CD41^+^ cells from E7.5–E 8.0 embryos. (**c**) Ery/P and non-Ery/P colonies generated per 3,000 sorted hemogenic endothelial cell populations negative for βH1-eGFP (n = 2). (**d**) Immunohistochemistry of E7.5 embryo for TIE2 (red), c-KIT (yellow), DAPI (Blue) and βH1-eGFP (green).

In order to extend and validate the results obtained *in vitro* using the ES cells to embryonic development *in vivo*, a transgenic βH1-eGFP mouse line was generated. Embryos were assessed at various developmental stages to define the spatio-temporal pattern of βH1-eGFP fluorescence (Fig. 5a). By E7.5, βH1-eGFP positive cells were detected within the ring formed by the blood islands in the yolk sac (marked with an arrow, Fig. 5a). By E8.0–E8.5, the yolk sac is in the process of encapsulating the embryo proper and the expression of βH1-eGFP was still restricted to the yolk sac area. Around the same developmental stage, heart beating and circulation are established and the primitive cells are disseminated into the vasculature. Accordingly, at the later E9.5 and E10.5 developmental stages, the expression of βH1-eGFP was observed within the vasculature of the whole embryo. Altogether these data confirm that the pattern of βH1-eGFP transgene detection is compatible with primitive erythrocytes homeostasis *in vivo*.

To determine whether *in vivo* primitive erythroid cells are also emerging from a βH1-eGFP^−^ HEII hemogenic endothelium, this immuno-phenotypic cell population was isolated from E7.5 βH1-eGFP embryos and replated in clonogenic assay (Fig. 5b). As shown in Fig. 5c, these cells generated mainly Ery/P primitive erythroid colonies as well as a few non-Ery/P colonies. These results suggest that competent primitive erythroid progenitors emerge directly from HEII and that the earliest blood progenitors generated by this cell population are primitive erythroid committed. To further document this relationship between HEII and βH1-eGFP *in vivo*, we performed immunohistochemistry analysis on E7.5 blood islands for βH1-eGFP as well as for TIE2 and c-KIT. We readily observed cells positive for these 3 markers further suggesting that primitive erythrocytes emerge from TIE2^+^ c-KIT^+^ hemogenic endothelial cells *in vivo* (Fig. 5d).

## Discussion

In this study we investigated the generation of primitive erythroid cells from hemangioblast and hemogenic endothelium using a new transgenic ES cell line and a mouse line in which eGFP is driven from the *βH1* globin regulatory sequences. With these new reporter lines, we established that primitive erythroid cells are generated from a hemogenic cell population similar to the one producing definitive hematopoietic cells. The relationship between primitive erythropoiesis and endothelium is further supported by a previous study that demonstrated that primitive erythroid cells emerge *in vivo* from extra-embryonic cells positive for endothelial markers^17^. We propose a model in which primitive erythrocytes are generated from hemangioblast *via* a hemogenic endothelium intermediate cell population. Although the *in vivo* existence of the hemangioblast in higher vertebrates as a precursor with both endothelial and hematopoietic potential has been recently experimentally questioned^25, 26^, there are conclusive evidences demonstrating that this precursor give rise *ex vivo* or *in vitro* to both lineages^8, 9, 27, 28^. This has led to the proposition that the hemangioblast is a state of competence which is never fulfilled *in vivo* due to the restriction toward a unique hematopoietic fate imposed by the microenvironment^29, 30^.

Our model of primitive erythroid blood cell development (Fig. 4c) is similar to the linear specification pathway model proposed for definitive hematopoiesis^13, 26^ in which mesodermal (hemangioblastic) precursors give rise to blood-committed hemogenic endothelium. Nonetheless Padron-Barthe *et al*.^26^ suggested that in contrast primitive erythroid and endothelial cells could be independently specified before gastrulation. However they might have missed an hemogenic endothelial intermediate stage as they performed their lineage tracing relatively late at E8.5 when most primitive erythroblasts have been already mostly generated and are starting to be dispersed in the vasculature.

Interestingly we observed that the hemogenic endothelium type 2 (HEII) cell population could be fractionated based on the expression of βH1-eGFP and that these two fractions displayed either mainly a Ery/P or non-Ery/P hematopoietic potential. These findings suggest that the commitment to primitive erythroid hematopoietic cell fate could be already established at the level of the hemogenic endothelium, suggesting the existence of hemogenic endothelium with specific hematopoietic potential. Although our study clearly indicates that primitive erythroid cells are generated from hemogenic endothelium cells, we cannot completely exclude that these cells could be also generated from other cellular sources, such as directly from the hemangioblast.

Previous reports have indicated that generation of primitive erythroid cells temporally precede the emergence of definitive blood cells in the mouse yolk sac^6^. This has lead to the concept of the existence of a first wave of generation of primitive hematopoietic cells followed by a second wave responsible for the production of EMPs that emerge independently from hematopoietic stem cells^4, 31–33^. Following studies demonstrated that EMPs emerge in the mouse embryos between E8.25 and E11 in the yolk sac vascular plexus first and then both yolk sac arterial and venous vasculature and that their production was associated with changes in morphologies consistent with an endothelial to hematopoietic transition (EHT)^34^. These results suggest that EMPs are generated from hemogenic endothelium embedded within the vasculature. In contrast, we detected hemogenic endothelium expressing βH1-eGFP within the blood islands at E7.5 before the presence of formed blood vessels. These results, as well as the *in vitro* results obtained with ES cells^13^, suggest that the EHT can proceed in the absence of inclusion of the hemogenic endothelium in the vasculature as well as in the absence of acquisition of an arterial fate. Although the spatiotemporal separation of emergence of primitive erythroid cells and EMPs in the embryos suggest the existence of hemogenic endothelium with restricted developmental potential, the results obtained with ES cells, and notably the presence of clonal blast colonies with both primitive and definitive potential^10^, suggest the existence of a common progenitor. Whether this is a hemangioblast or a hemogenic endothelium precursor that becomes restricted to a specific cell fate by spatiotemporal clues from the environment *in vivo*, remains to be determined.

We previously demonstrated that the two transcriptional repressors GFI1 and GFI1b, that are transcriptional targets of RUNX1, are critical for the EHT process as they are driving the down-regulation of the endothelial program during this transition and the changes in morphology associated with the EHT^35–37^. The observation that primitive erythroid cells are still present in the vasculature in absence of RUNX1^38–40^ is therefore contradictory to their generation from an hemogenic endothelium by EHT. We propose that the residual expression of *Gfi1b* detected in the absence of RUNX1^35^ could be sufficient to specifically support the release in the bloodstream of primitive erythroid cells. Why this is not also resulting in the emergence of definitive blood cells remain to be elucidated.

In conclusion, our study shed new light on the emergence of primitive erythrocytes. Our study formally demonstrates that primitive erythroid cells are generated from a hemogenic endothelium. The absence of inclusion of this precursor within mature vessels suggests that this precursor could be more appropriately, as previously proposed^12, 41^, defined as an hemogenic angioblast. A fascinating goal will be to try to understand the relationship and difference between the different hemogenic endothelium giving rise to specific blood lineages. The knowledge acquired by these studies would expand our understanding of the process of blood cell generation at the cellular and molecular levels^42^ and could be potentially applied in the future to generate specific types of blood cells for cell-replacement therapies.

## Materials and Methods

### BAC Recombineering

The bMQ433i10 BAC was purchased from the Sanger Institute. SW105 bacterial strain containing λ Red prophage expressing recombination proteins: *exo*, *bet* and *gam* under the control of temperature-sensitive repressor was used^43, 44^. The homology arms were cloned into a vector containing: eGFP reporter gene, a floxed cassette PGK/EM7-TK-Neo and an ampicillin resistance gene. Recombineering was performed according to the method previously described^45^.

### Gene Expression Analysis

Total RNA was extracted using RNeasy Plus Mini Kit (Qiagen). cDNA was generated using Omniscript RT Kit (Qiagen). Quantitative PCR was performed on ABI7900 (Applied Biosystems) using universal probe library (Roche) and TaqMan Universal PCR Master Mix (Applied Biosystems). Gene expression was normalised to a reference gene (β-Actin) and represented as 2^−ΔCt^.

### ES cell culture

Murine ES cells were maintained and differentiated as described previously^46^. Hemogenic endothelial cultures were performed as previously described^13^.

### Generation of endothelial networks

ES derived cells were seeded on the OP9 stromal cell layer in the medium containing MEMα (Gibco) supplemented with 20% FBS serum (PAA Laboratories). Fresh media was added every three days and cells were cultured for 10–14 days. The endothelial networks were subsequently stained with antibodies.

### Cells Immunohistochemistry

Cells were washed with 1x PBS and then incubated for 5 minutes at 4 °C in PBS supplemented with 20% FBS (PAA Laboratories). Next, cells were stained with PECAM-1 antibody (BD-Pharmingen) in IMDM medium supplemented with 10% FBS and incubated at 4 °C for no less than 30 minutes. Following incubations, cells were washed three times with 1x PBS. Next, cells were stained with secondary antibody, Streptavidin-Alexa-555 and incubated at 4 °C for no less than 30 minutes. Following this incubation, cells were washed three times with 1x PBS and visualised directly under the microscope.

### Fluorescence-activated cell sorting (FACS)

Flk1-bio, TIE2-bio, CD31-bio, c-KIT-APC, VE-CADHERIN-APC, CD41-PE and Streptavidin-PECy7 antibodies were purchased from eBioscience. The stained cells were sorted on ARIA or Influx flow cytometers or analysed on a FACS LSRII (Becton Dickinson). The staining data were analysed using the FlowJo software (TreeStar).

### Live cell imaging

Cells were seeded at a density of 8.0 × 10^5^–1.2 × 10^5^ cells/cm^2^ into either multiwell tissue culture plates (BD Falcon) or into Ibidi μ-slides, in the appropriate culture media. Cells were placed in the INCUCYTE^TM^ Live-Cell Imaging System (Essen Bioscience). Images were acquired every 5–30 minutes, depending on the experiment. Data was exported using INCUCYTE^TM^ software, provided by the manufacturer.

### Generation of transgenic mouse lines

Following microinjections into blastocyst and reimplantations, pups with the highest level of chimerism were selected and crossed with C57BL/6 mice to generate agouti progeny. The presence of the transgene was validated by PCR and transgenic agouti mice were further crossed with C57BL/6 mice to generate subsequent generations. All animal work was performed under regulation governed by the Home Office Legislation under the Animal Scientific procedures Act (ASPA) 1986 and was approved by the Animal Welfare and Ethics Review Body (AWERB) of the Cancer Research UK Manchester Institute.

### Embryo generation and dissection

Timed matings were set overnight between sets of transgenic βH1-eGFP male and two wild-type ICR females. The presence of vaginal plugs was assessed the following morning. The day in which the plug was found was considered E0.5. The exact developmental stage of embryos was assessed by somite counts and morphological features^47^. The dissected tissues were trypsinised, single cell suspensions were filtered through Filcon (Becton Dickinson) and further characterised by FACS analysis or tissue culture.

### Embryos Immunohistochemistry

Embryos still in decidua were fixed in 4% Paraformaldehyde (PFA) for two hours, before they were soaked in 30% sucrose and mounted in OCT compound. 7 µm sections were prepared using a cryostat. The sections were streptavidin/biotin blocked followed by serum blocking (PBS with 10% FCS, 0.05% Tween20 and of 10% goat serum (DAKO) for 1 hour before the sections were incubated with primary antibodies at 4 °C overnight in blocking buffer. Primary antibodies used were rabbit anti –GFP (598, polyclonal, MBL) (1/200); rat anti-mouse c-kit (553352, clone 2B8, BD Biosciences) (1/100); biotinylated rat anti-mouse Tie2 (13–5987, clone TEK4 eBioscience) (1/100). After staining, the sections were washed three times in PBST for 15 minutes each and then incubated with fluorochrome-conjugated secondary antibody at room temperature for 1 hour. These were Alexa Fluor® 488 Donkey Anti-Rabbit IgG (H + L) (A21206, Life Technologies); Alexa Fluor® 647 Goat Anti-Rat IgG (H + L) (A21247, Life Technologies); Streptavidin, Alexa Fluor® 555 Conjugate (S32355, Life Technologies). All secondary antibodies were used at 1/400 dilution. The sections were then further washed three times in PBS and mounted using Prolong Gold anti-fade medium with DAPI (Life Technologies). Images were taken using a low-light time lapse microscope (Leica) using the Metamorph imaging software and processed using ImageJ.

### Statistical analysis

Statistical analysis was performed using Student’s t test. Significant differences are indicated with *** if p < 0.001.

## Electronic supplementary material

Supplementary Information

Supplementary video
