# Supplementary material for: Primitive erythrocytes are generated from hemogenic endothelial cells
Source: Sci Rep. 2017 Jul 25;7:6401. doi: 10.1038/s41598-017-06627-9 (PMC5526883; doi:10.1038/s41598-017-06627-9)
Supplement: Supplementary file 1 — Supplementary Information [file 41598_2017_6627_MOESM1_ESM.pdf]

Supplementary Information:

Primitive erythrocytes are generated from hemogenic endothelial cells

Monika Stefanska, Kiran Batta, Rahima Patel, Magdalena Florkowska, Valerie Kouskoff & Georges Lacaud

Supplementary Figure legends:

Figure S1: (a) Screen capture of the genomic elements included in the BAC bMQ433I10. The BAC bMQ433I10 contains the entire murine β globin locus. **(b)** Percentage of βH1-eGFP positive cells at indicated days during EB differentiation. Data represented are means ± SD (n=4). **(c)** Percentage of populations expressing different levels of βH1-eGFP (P0-P3) in EBs at day 5 of differentiation. Data represented are means ± SD (n=4). **(d)** Populations expressing different levels of βH1-eGFP were sorted (P0-P3) from day 5 EBS. FACS analysis of βH1-eGFP expression in before and after sort purification.

Figure S2: (a-c) βH1-eGFP positive cells were sorted from day 5 EBs and cultured in either Erythropoietin (Epo) or Epo and Kit-Ligand (Epo + KL) containing media. Day 5 EBs were used as a control. **(a)**. Number of Ery/P or non-Ery/P colonies generated following replating of 40,000 βH1-eGFP positive cells in indicated conditions. Data represented are means ± SD from one representative experiment (n=3) **(b)** Representative images of CFUs observed following replating of indicated cells in indicated conditions **(c)**. *O*-dianisidine and May-Grunwald Giemsa staining of cytospin preparations of βH1-eGFP positive cells cultured in indicated conditions **(d)**. *O*-dianisidine and May-Grunwald Giemsa stained cytospin preparations of hand picked of Ery/P or non-Ery/P colonies. Scale bar 50 μM. **(e)**. Gene expression analyses of globin genes with respect to β-actin in βH1-eGFP positive cells cultured in indicated conditions. Expressions of embryonic hemoglobin α-chain (ζ) and β-chains (β-H1 and εy) and expressions of adult hemoglobin α-chain (α) and β-chains (β1-βmajor, β2-βminor) were analysed. Please note that primitive red cells can express both embryonic and adult hemoglobin

genes whereas definitive erythroid cells express only adult hemoglobin genes. Data represented are means $\pm$ SD from one representative experiment (n=3)

Figure S3: **(a)** Percentage of βH1-eGFP positive cells at indicated days during blast colony differentiation. Data represented are means $\pm$ SD (n=4). (**b)** Percentage of indicated cell populations at indicated days during blast colony differentiation. Data represented are mean $\pm$ SD (n=4).

Figure S4: BL-CFCs were sorted from day 3.25 EBs and further cultured in liquid blast media for 42 hours. **(a)** Percentage of HEI and HEII in βH1-eGFP^{-} c-KIT^{+} cells. Data represented are mean $\pm$ SD (n=4). **(b)** Percentage of βH1-eGFP^{+} cells observed in HEI and HEII cell populations after culture in liquid blast media. Data represented are means $\pm$ SD (n=6). (**c)** Phase contrast images of βH1-eGFP negative TIE2/c-KIT double positive cells cultured on gelatin in hematopoietic conditions for the indicated days. (d) Percentage of HEII cells positive or negative for βH1-eGFP after culture in liquid blast media. Data represented are means $\pm$ SD (n=3).

Supplementary Video 1:

Bright field and fluorescence time-lapse imaging of emergence of βH1-eGFP positive cells during *in vitro* blast colony differentiation.

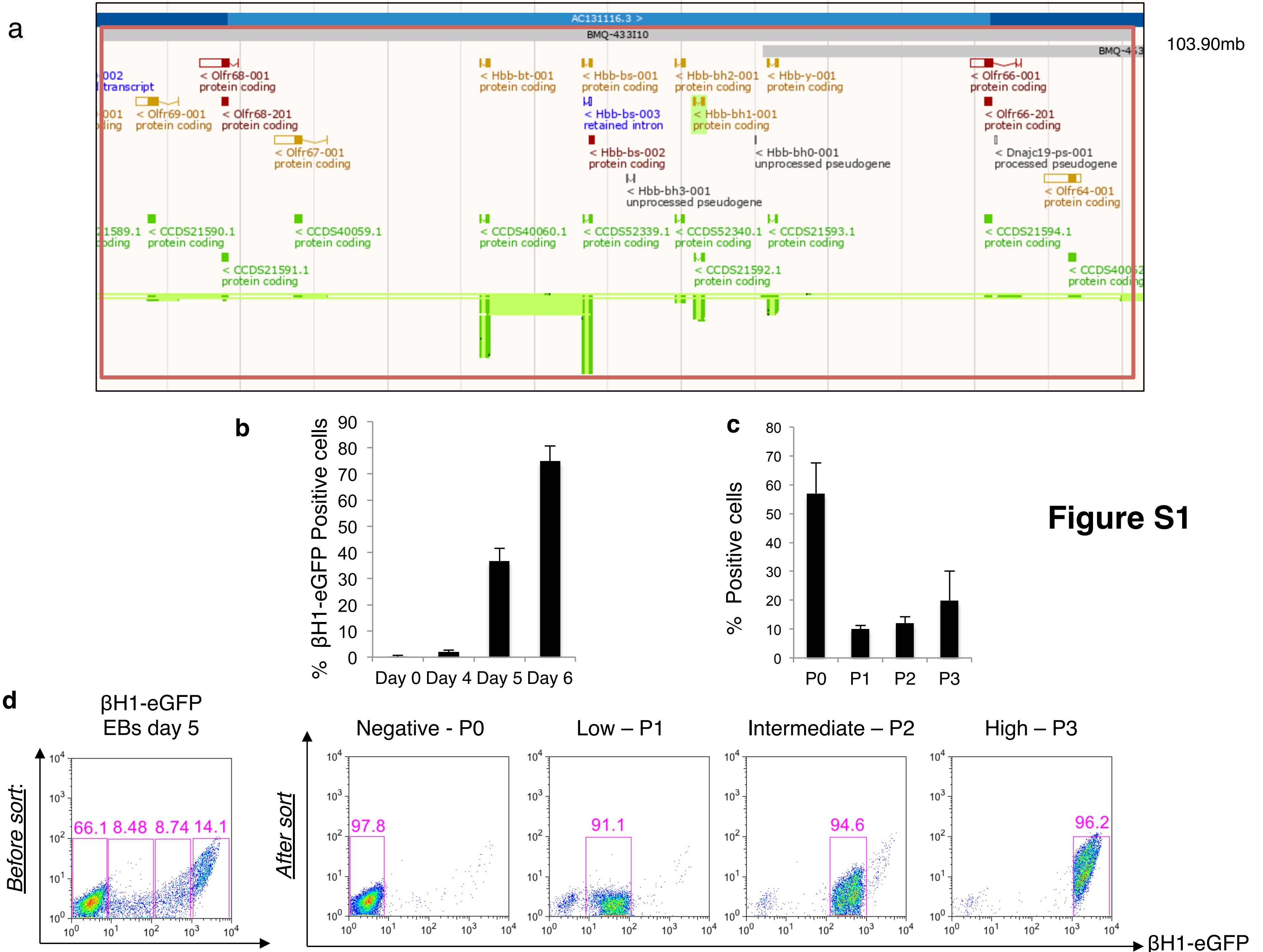

a
AC131116.3 >
BMQ-433I10
103.90mb
< Olfr68-001
protein coding
< Olfr69-001
protein coding
< Olfr68-201
protein coding
< Olfr67-001
protein coding
< Hbb-bt-001
protein coding
< Hbb-bs-001
protein coding
< Hbb-bs-003
retained intron
< Hbb-bs-002
protein coding
< Hbb-bh3-001
unprocessed pseudogene
< Hbb-bh2-001
protein coding
< Hbb-bh1-001
protein coding
< Hbb-bh0-001
unprocessed pseudogene
< Hbb-y-001
protein coding
< Olfr66-001
protein coding
< Olfr66-201
protein coding
< Dnajc19-ps-001
processed pseudogene
< Olfr64-001
protein coding
< CCDS21590.1
protein coding
< CCDS21591.1
protein coding
< CCDS40059.1
protein coding
< CCDS40060.1
protein coding
< CCDS52339.1
protein coding
< CCDS52340.1
protein coding
< CCDS21592.1
protein coding
< CCDS21593.1
protein coding
< CCDS21594.1
protein coding
b
% βH1-eGFP Positive cells
Day 0
Day 4
Day 5
Day 6
c
% Positive cells
P0
P1
P2
P3
d
βH1-eGFP
EBs day 5
Before sort
66.1
8.48
8.74
14.1
After sort
Negative - P0
97.8
Low – P1
91.1
Intermediate – P2
94.6
High – P3
96.2
βH1-eGFP

Figure S1

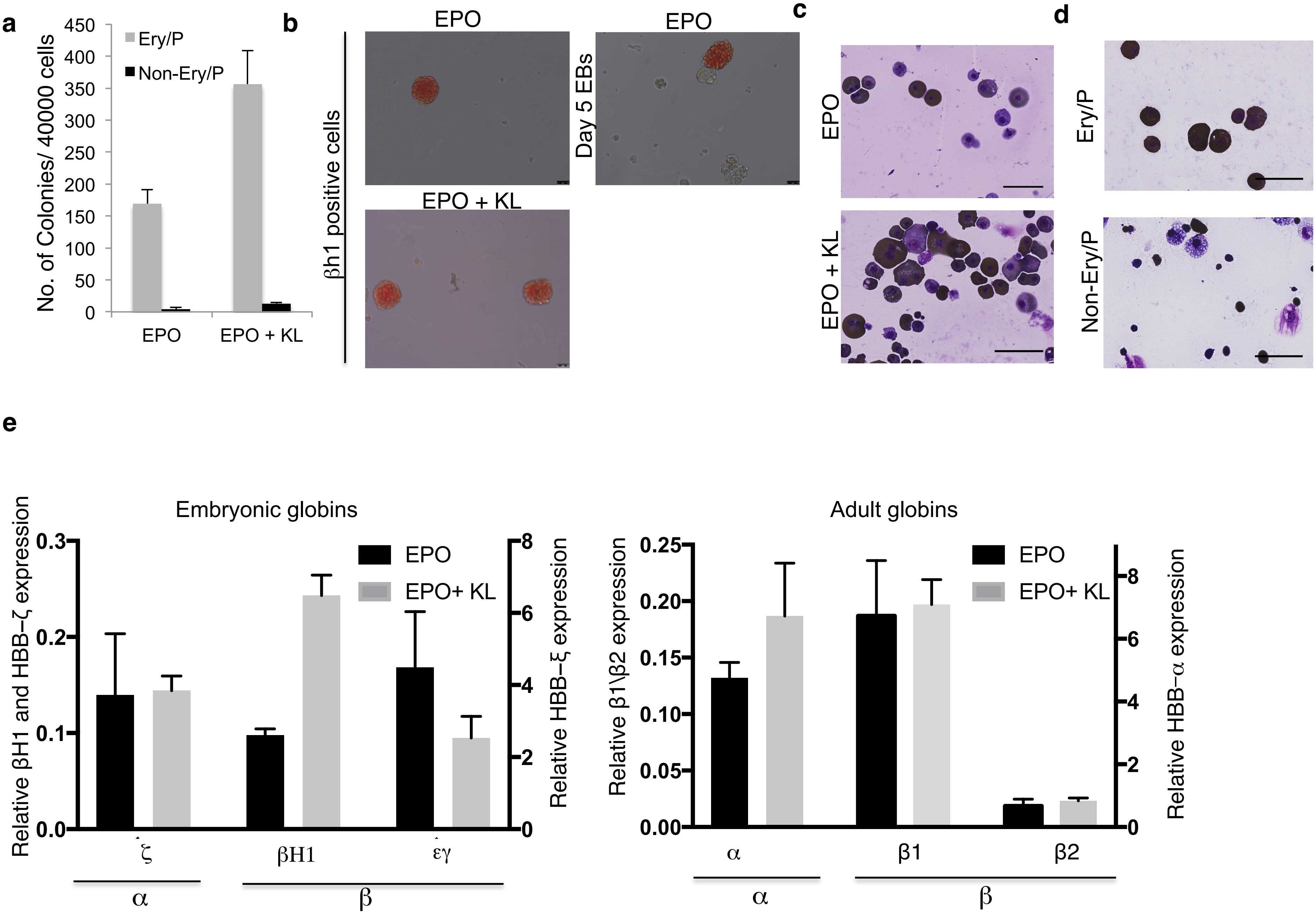
a
No. of Colonies/ 40000 cells
Ery/P
Non-Ery/P
450
400
350
300
250
200
150
100
50
0
EPO
EPO + KL
b
βh1 positive cells
EPO
EPO + KL
Day 5 EBs
EPO
c
EPO
EPO + KL
d
Ery/P
Non-Ery/P
e
Embryonic globins
Relative βH1 and HBB-ζ expression
Relative HBB-ξ expression
EPO
EPO+ KL
0.3
0.2
0.1
0.0
8
6
4
2
0
ζ
βH1
εγ
α
β
Adult globins
Relative β1\β2 expression
Relative HBB-α expression
0.25
0.20
0.15
0.10
0.05
0.00
α
β1
β2

Figure S2

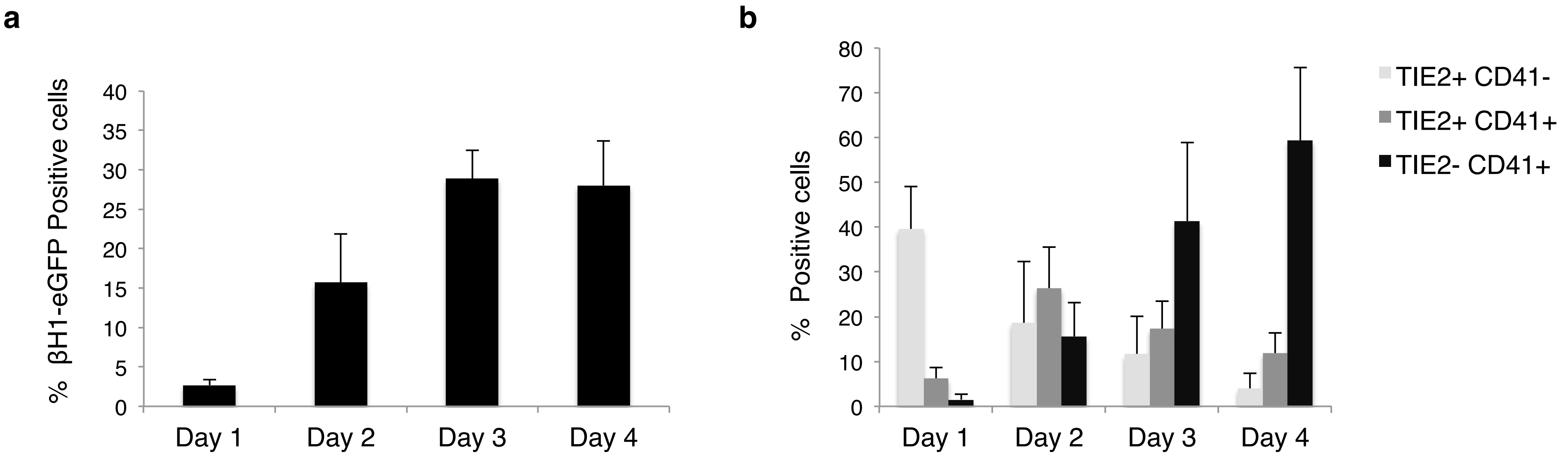
a
% βH1-eGFP Positive cells
40
35
30
25
20
15
10
5
0
Day 1
Day 2
Day 3
Day 4
b
% Positive cells
80
70
60
50
40
30
20
10
0
Day 1
Day 2
Day 3
Day 4
TIE2+ CD41-
TIE2+ CD41+
TIE2- CD41+

Figure S3

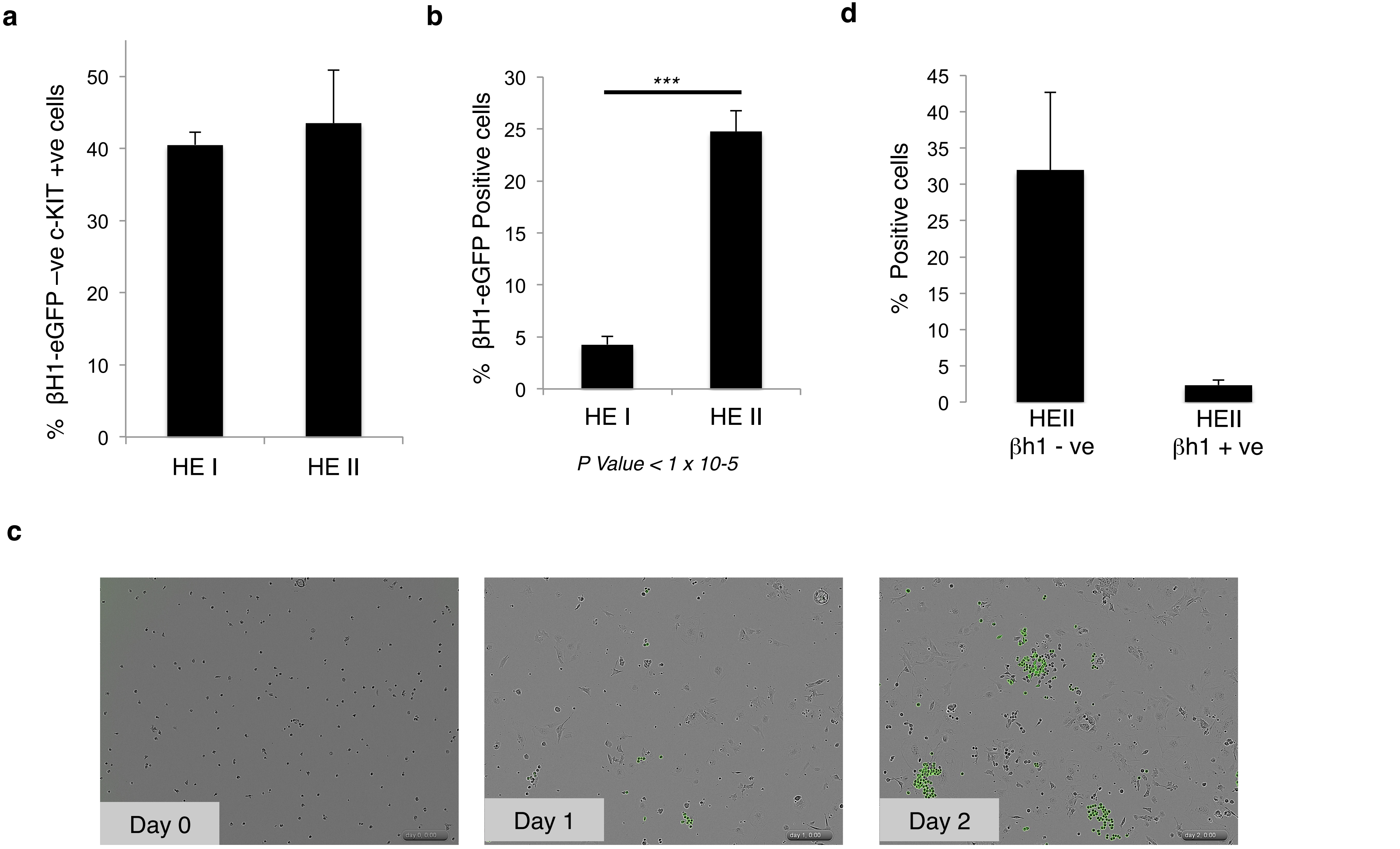
a
% βH1-eGFP –ve c-KIT +ve cells
0
10
20
30
40
50
HE I
HE II
b
% βH1-eGFP Positive cells
0
5
10
15
20
25
30
***
HE I
HE II
P Value < 1 x 10-5
d
% Positive cells
0
5
10
15
20
25
30
35
40
45
HEII
βh1 - ve
HEII
βh1 + ve
c
Day 0
Day 1
Day 2

Figure S4
